# Pneumothorax During Cervical Paraspinal Muscle Electromyography: A Case Report

**DOI:** 10.7759/cureus.2927

**Published:** 2018-07-05

**Authors:** Muniba Fayyaz, Rollin J Hawley

**Affiliations:** 1 Internal Medicine, Fatima Memorial Hospital, Lahore, PAK; 2 Neurology, Carilion New River Valley Medical Center, Pulaski, USA

**Keywords:** electromyography, complication, pneumothorax, paraspinal

## Abstract

Needle electromyography is a common procedure used by physicians to detect an underlying neuromuscular disorder. It is performed by inserting a needle electrode in different locations of the muscles and measures the muscle reaction or electrical activity in response to a nerve's stimulation of the muscle on an oscilloscope. The procedure is usually safe, however, in rare cases, the insertion of the needle in the pleura or lung can cause a pneumothorax, which can be life-threatening. Here, we have reported a case in which a puncture in the right cervical paraspinal muscle during a needle electromyography procedure caused a right-sided pneumothorax in a 49-year-old female patient who was further treated in the hospital until the pneumothorax resolved. In addition, a technique that can be used to prevent this life-threatening complication will also be discussed. To our knowledge, one similar case report has been presented in the past.

## Introduction

A pneumothorax is defined as air within the pleural cavity [1]. Its incidence during an electromyography (EMG) procedure is very rare, but its occurrence can be life-threatening. While conducting an EMG, the following sites have been reported in previous pneumothorax cases: the cervical root, Erb’s point, diaphragm, serratus anterior, supraspinatus, paracervical, rhomboid, and intercostal muscles [2-6]. Presented here is the case report of a patient who experienced a pneumothorax after needle insertion into her right cervical paraspinal muscle.

## Case presentation

A 49-year-old woman presented to our neurology clinic with complaints of pain and weakness in her upper-right and lower-left extremities, lower back pain, and numbness in her lower extremities for many years. For the past couple of months, the pain in her lower back, left hip, and lower extremities (including feet) has gotten worse. The pain increased at night, was exacerbated while standing or sitting, and was accompanied by numbness in her left lateral thigh. The rest of her medical history was unremarkable. The nerve conduction studies showed mild but painful sensory axonal neuropathy with superimposed mild bilateral sensory carpal tunnel syndrome. During the needle EMG using a 50 mm * 25 gauge needle, she complained of a significant amount of discomfort when her right cervical paraspinal muscles were punctured; however, she did not exhibit any other symptoms. After the EMG study, her blood pressure was 156/103 mmHg, with a pulse rate of 90/min (right radial, sitting), then it was 154/101 mmHg with a pulse rate of 97/min. She was given the appropriate treatment, which included blood tests to eliminate correctable causes of neuropathy, vitamin B12, exercise, and appropriate medications.

After leaving the clinic symptom-free, she returned 30 minutes later, with complaints of right-sided pleuritic chest pain and the coughing up of some mucus. Upon physical examination, she had a tender right pectoralis major muscle, equal breath sounds bilaterally, a normal cardiac examination, a blood pressure of 130/80 mmHg, and a pulse of 100/min (right brachial, sitting). She was immediately sent to the emergency room for a workup, including a chest X-ray, which disclosed a 15% right-sided pneumothorax. This was most likely due to the needle EMG puncture of her right cervical paraspinal muscles. The patient was admitted overnight for non-rebreathing oxygen treatment. Once her symptoms were resolved, she was discharged with a follow-up to ensure that her pneumothorax had resolved.

## Discussion

Based on this case report, the lung tissue is susceptible to damage during an EMG procedure, which can eventually lead to a life-threatening condition. Although a pneumothorax is rare during an EMG procedure, it can occur, and the examiner should keep this complication in mind while performing an EMG of the cervical paraspinal muscles.

Honet et al. presented a similar case report in which a needle puncture of the paracervical muscles produced a pneumothorax [3]. The pneumothorax was 20% in their case, while it was 15% in ours [3]. It is beneficial to know the anatomy of the region to gain a better understanding and avoid future complications. Cadavers and cervical spine radiographs were used to assess the lung tissue vulnerability to paracervical muscle needle insertion in their study. Using the cervical spine radiographs, the authors determined the depth and location of the underlying lung tissue, and they eventually demonstrated that five out of 23 patient radiographs had lung tissue extending above the clavicles, with a distance from the skin surface to the lung tissue of around 3.3 cm [3]. This can allow the standard 37-mm EMG needle electrode to penetrate the lung [3]. In our case, a 50-mm needle was used, which could have easily penetrated the lung pleura. Therefore, the authors’ recommended that when an examiner encounters a patient with a long and thin neck (with more cervical vertebrae above the clavicle and, thus, more susceptible), an auscultation of the neck should be done to determine if the lung tissue is within the examination field. They also recommended that the needle electrode be inserted close to the midline and dorsal to the transverse process. Additionally, the audio should be turned on in order to monitor the electrical activity throughout the procedure, as this can help the examiner determine if the needle is placed in the correct location and avoid the possibility of inserting the needle into the lung/pleura [3]. An ultrasound of the neck can also be a possible method used for the detection of lung tissue in the examination field in addition to auscultation [7].

Several other studies have reported the same complication involving different muscles. For example, Kassardjian et al. conducted a study demonstrating that out of 64,490 EMG procedures, only seven patients presented with a pneumothorax. The serratus anterior and diaphragm were the muscles most commonly involved in producing the pneumothorax [1]. Reinstein et al. reported a case in which the supraspinatus muscle was the origin of the pneumothorax during an EMG procedure. They explained that the supraspinatus muscle overlies the pleural cavity; therefore, its puncture is more likely to cause a pneumothorax. In cervical radiculopathy and Erb’s palsy cases, they recommended the needle electrode insertion of the infraspinatus muscle because it is three times larger in size than the supraspinatus muscle and further from the pleural cavity. Therefore, there is a lower chance of causing a pneumothorax. However, in suprascapular nerve entrapment cases, a needle insertion into both muscles three-quarters of the distance from the acromion to the vertebral border (where the supraspinatus fossa is the widest) is recommended [2].

In addition, Miller presented a case in which an EMG study of the left rhomboid major muscle was the cause of the pneumothorax. It was emphasized that the patient's history of neck surgery for cancer could have produced anatomical distortions and fibrosis that affected the pleura, making it more vulnerable to producing a pneumothorax [4]. Finally, Sander et al. reported a case in which cervical root stimulation produced a pneumothorax. This could have been because of the needle insertion to a position abutting the lateral vertebral arch, which is adjacent to the pleura. Instead of an angled lateral needle insertion, a straight insertion of the needle was recommended. They also suggested that a visual and auditory analysis, as well as connecting the electrode to the stimulator for data acquisition during insertion, could help prevent a pneumothorax [5].

## Conclusions

Pneumothorax has been reported in the past involving different muscles during an EMG procedure, and different techniques have been recommended to prevent this type of complication. Although a pneumothorax is a rare finding, constant awareness is still required while performing EMG procedures in order to avoid this life-threatening condition.
